# An alternative error bound for linear complementarity problems involving $B^{S}$-matrices

**DOI:** 10.1186/s13660-018-1618-x

**Published:** 2018-02-02

**Authors:** Lei Gao

**Affiliations:** School of Mathematics and Information Science, Baoji University of Arts and Sciences, Baoji, P.R. China

**Keywords:** 65G50, 90C31, 90C33, Error bounds, Linear complementarity problems, $B^{S}$-matrices, *P*-matrices

## Abstract

An alternative error bound for linear complementarity problems for $B^{S}$-matrices is presented. It is shown by numerical examples that the new bound is better than that provided by García-Esnaola and Peña (Appl. Math. Lett. 25(10):1379–1383, 2012) in some cases. New perturbation bounds of $B^{S}$-matrices linear complementarity problems are also considered.

## Introduction

The linear complementarity problem is to find a vector $x\in \mathbb{R}^{n}$ such that

1 $$ x\geq0,\qquad Mx+q\geq0,\qquad (Mx+q)^{T}x=0, $$

where $M\in\mathbb{R}^{n\times n}$ and $q\in\mathbb{R}^{n}$. We denote problem (1) and its solution by $\operatorname{LCP}(M, q)$ and $x^{*}$, respectively. The $\operatorname{LCP}(M, q)$ often arises from the various scientific areas of computing, economics and engineering such as quadratic programs, optimal stopping, Nash equilibrium points for bimatrix games, network equilibrium problems, contact problems, and free boundary problems for journal bearing, etc. For more details, see [2–4].

An interesting problem for the $\operatorname{LCP}(M, q)$ is to estimate

2 $$ \max_{d\in[0,1]^{n}}\big\| (I-D+DM)^{-1} \big\| _{\infty}, $$

since it can often be used to bound the error $\|x-x^{*}\|_{\infty}$ [5], that is,

$$ \big\| x-x^{*}\big\| _{\infty} \leq\max_{d\in[0,1]^{n}}\big\| M_{D}^{-1} \big\| _{\infty} \big\| r(x)\big\| _{\infty}, $$

where $M_{D}=I-D+DM$, $D=\operatorname{diag}(d_{i})$ with $0\leq d_{i} \leq1$ for each $i\in N$, $d=[d_{1},d_{2},\ldots,d_{n}]^{T}\in[0,1]^{n}$, and $r(x)=\min\{ x,Mx+q\}$ in which the min operator denotes the componentwise minimum of two vectors; for more details, see [1, 6–14] and the references therein.

In [1], García-Esnaola and Peña provided an upper bound for (2) when *M* is a $B^{S}$-matrix as a subclass of *P*-matrices [15], which contains *B*-matrices. Here a matrix $M=[m_{ij}]\in\mathbb{R}^{n\times n}$ is called a *B*-matrix [16] if, for each $i\in N=\{1,2,\ldots,n\}$,

$$ \sum_{\substack{k\in N}}m_{ik}>0,\quad \text{and}\quad \frac{1}{n} \biggl(\sum_{\substack{k\in N}}m_{ik} \biggr)>m_{ij} \quad\text{for any } j\in N \text{ and } j\neq i, $$

and a matrix $M=[m_{ij}]\in\mathbb{R}^{n\times n}$ is called a $B^{S}$-matrix [15] if there exists a subset *S*, with $2\leq \operatorname{card}(S)\leq n-2$, such that, for all $i,j\in N$, $t\in T(i)\setminus\{i\}$, and $k\in K(j)\setminus\{j\}$,

$$R_{i}^{S}>0, R_{j}^{\overline{S}}>0,\quad \text{and}\quad \bigl(m_{it}-R_{i}^{S} \bigr) \bigl(m_{jk}-R_{j}^{\overline{S}} \bigr)< R_{i}^{\overline{S}}R_{j}^{S}, $$

where $R_{i}^{S}=\frac{1}{n}\sum_{\substack{k\in S}}m_{ik}$, $T(i):=\{t\in S | m_{it}>R_{i}^{S}\}$ and $k(j):=\{k\in \overline{S} | m_{jk}>R_{j}^{\overline{S}}\}$ with $\overline{S}=N\setminus\{S\}$.

### Theorem 1

([1, Theorem 2.8])

*Let* $M=[m_{ij}]\in\mathbb{R}^{n\times n}$ *be a* $B^{S}$-*matrix*, *and let* $X=\operatorname{diag}(x_{1},x_{2},\ldots,x_{n})$ *with*

$$x_{i}=\left \{ \textstyle\begin{array}{l@{\quad}l} \gamma, &i\in S,\\ 1, &otherwise, \end{array}\displaystyle \right . $$

*such that* $\tilde{M}:=MX$ *is a* *B*-*matrix with the form* $\tilde{M}=\tilde{B}^{+}+\tilde{C}$, *where*

3 $$ \tilde{B}^{+} =[\tilde{b}_{ij}]= \left [ \textstyle\begin{array}{c@{\quad}c@{\quad}c} m_{11}x_{1}-\tilde{r}_{1}^{+} &\cdots &m_{1n}x_{n}-\tilde{r}_{1}^{+} \\ \vdots & &\vdots \\ m_{n1}x_{1}-\tilde{r}_{n}^{+} &\cdots &m_{nn}x_{n}-\tilde{r}_{n}^{+} \end{array}\displaystyle \right ],\qquad \tilde{C}=\left [ \textstyle\begin{array}{c@{\quad}c@{\quad}c} \tilde{r}_{1}^{+} &\cdots &\tilde{r}_{1}^{+} \\ \vdots & &\vdots \\ \tilde{r}_{n}^{+} &\cdots &\tilde{r}_{n}^{+} \end{array}\displaystyle \right ], $$

*and* $\tilde{r}_{i}^{+}=\max\{ 0,m_{ij}x_{j} | j\neq i\}$. *Then*

4 $$ \max_{d\in[0,1]^{n}}\big\| M_{D}^{-1} \big\| _{\infty} \leq \frac{(n-1)\max\{\gamma,1\}}{\min\{\tilde{\beta},\gamma,1\}}, $$

*where* $\tilde{\beta}=\min_{i\in N}\{\tilde{\beta}_{i}\}$ *with* $\tilde{\beta}_{i}=\tilde{b}_{ii}-\sum_{j\neq i}|\tilde{b}_{ij}|$, *and*

5 $$ (0< )\ \gamma\in \biggl(\max_{j\in N,k\in K(j)\setminus \{j\}}\frac{m_{jk}-R_{j}^{\overline{S}}}{R_{j}^{S}}, \min_{\substack{i\in N,t\in T(i)\setminus \{i\}}}\frac{R_{i}^{\overline{S}}}{m_{it}-R_{i}^{S}} \biggr), $$

*where* max (min) *is set to be* −∞ (∞) *if* $K(j)\setminus \{j\}=\emptyset$ ($T(i)\setminus\{i\}=\emptyset$).

Note that for some $B^{S}$ matrices, *β̃* can be very small, thus the error bound (4) can be very large (see examples in Section 3). Hence it is interesting to find an alternative bound for $\operatorname{LCP}(M, q)$ to overcome this drawback. In this paper we provide a new upper bound for (2) and give a family of examples of $B^{S}$-matrices that are not *B*-matrices for which our bound is a small constant in contrast to bound (4) of [1], which can be arbitrarily large. Particularly, when the involved matrix is a *B*-matrix as a special class of $B^{S}$-matrices, the new bound is in line with that provided by Li *et al.* in [13].

## Main result

First, recall some definitions and lemmas which will be used later. A matrix $M=[m_{ij}]\in\mathbb{R}^{n\times n}$ is called: (1) a *P*-matrix if all its principal minors are positive; (2) a strictly diagonally dominant (*SDD*) matrix if $|m_{ii}|>\sum_{j\neq i}^{n}|m_{ij}|$ for all $i=1,2,\ldots,n$; (3) a nonsingular *M*-matrix if its inverse is nonnegative and all its off-diagonal entries are nonpositive [2].

### Lemma 1

([1, Theorem 2.3])

*Let* $M=[m_{ij}]\in\mathbb{R}^{n\times n}$ *be a* $B^{S}$-*matrix*. *Then there exists a positive diagonal matrix* $X=\operatorname{diag}(x_{1},x_{2},\ldots,x_{n})$ *with*

$$x_{i}=\left \{ \textstyle\begin{array}{l@{\quad}l} \gamma, &i\in S,\\ 1, &otherwise, \end{array}\displaystyle \right . $$

*such that* $\tilde{M}:=MX$ *is a* *B*-*matrix*.

### Lemma 2

([1, Lemma 2.4])

*Let* $M=[m_{ij}]\in \mathbb{R}^{n\times n}$ *be a* $B^{S}$-*matrix*, *and let* *X* *be the diagonal matrix of Lemma* 1 *such that* $\tilde{M}:=MX$ *is a* *B*-*matrix with the form* $\tilde{M}=\tilde{B}^{+}+\tilde{C}$, *where* $\tilde{B}^{+}=[\tilde{b}_{ij}]$ *is the matrix of* (3). *Then* $\tilde{B}^{+}$ *is strictly diagonally dominant by rows with positive diagonal entries*.

### Lemma 3

([1, Lemma 2.6])

*Let* $M=[m_{ij}]\in \mathbb{R}^{n\times n}$ *be a* $B^{S}$-*matrix that is not a* *B*-*matrix*, *then there exist* $k,i\in N$ *with* $k\neq i$ *such that*

6 $$ m_{ik}\geq\frac{1}{n}\sum _{j=1}^{n}m_{ij}. $$

*Furthermore*, *if* $k\in S$ (*resp*., $k\in\overline{S}$), *then* $\gamma<1$ (*resp*., $\gamma>1$), *where the parameter* *γ* *satisfies *(5).

Lemma 3 will be used in the proof of Corollary 1.

### Lemma 4

[17, Theorem 3.2] *Let* $A=[a_{ij}]$ *be an* $n\times n$ *row strictly diagonally dominant* *M*-*matrix*. *Then*

$$ \big\| A^{-1}\big\| _{\infty}\leq\sum_{i=1}^{n} \Biggl(\frac{1}{a_{ii}(1-u_{i}(A)l_{i}(A))}\prod_{j=1}^{i-1} \frac {1}{1-u_{j}(A)l_{j}(A)} \Biggr), $$

*where* $u_{i}(A)=\frac{1}{|a_{ii}|}\sum_{j=i+1}^{n}|a_{ij}|$, $l_{k}(A)=\max_{k\leq i\leq n} \{\frac{1}{|a_{ii}|}\sum_{\small\substack{j=k,\\j\neq i}}^{n}|a_{ij}| \}$, *and* $\prod_{j=1}^{i-1}\frac{1}{1-u_{j}(A)l_{j}(A)}=1$ *if* $i=1$.

### Lemma 5

([12, Lemma 3])

*Let* $\gamma> 0$ *and* $\eta\geq0 $. *Then*, *for any* $x\in[0,1]$,

$$\frac{1}{1-x+\gamma x} \leq \frac{1}{\min\{\gamma,1\}} $$

*and*

$$\frac{\eta x}{1-x+\gamma x} \leq\frac{\eta}{\gamma}. $$

### Lemma 6

([11, Lemma 5])

*Let* $A=[a_{ij}]\in\mathbb{R}^{n\times n}$ *with* $a_{ii}>\sum_{j=i+1}^{n}|a_{ij}|$ *for each* $i\in N$. *Then*, *for any* $x_{i}\in[0,1]$,

$$ \frac{1-x_{i}+a_{ii}x_{i}}{1-x_{i}+a_{ii}x_{i}-\sum_{j=i+1}^{n}|a_{ij}|x_{i}} \leq \frac{a_{ii}}{a_{ii}-\sum_{j=i+1}^{n}|a_{ij}|}. $$

We now give the main result of this paper by using Lemmas 1, 2, 4, 5, and 6.

### Theorem 2

*Let* $M=[m_{ij}]\in\mathbb{R}^{n\times n}$ *be a* $B^{S}$-*matrix and* $X=\operatorname{diag}(x_{1},x_{2},\ldots,x_{n})$ *with*

$$ x_{i}=\left \{ \textstyle\begin{array}{l@{\quad}l} \gamma, &i\in S,\\ 1, &otherwise, \end{array}\displaystyle \right . $$

*such that* $\tilde{M}:=MX$ *is a* *B*-*matrix with the form* $\tilde{M}=\tilde{B}^{+}+\tilde{C}$, *where* $\tilde{B}^{+}=[\tilde{b}_{ij}]$ *is the matrix of* (3). *Then*

7 $$ \max_{d\in[0,1]^{n}}\big\| M_{D}^{-1} \big\| _{\infty} \leq \sum_{i=1}^{n} \frac{(n-1)\max\{\gamma,1\}}{\min\{\widehat{\beta }_{i},x_{i}\}}\prod_{j=1}^{i-1} \frac{\tilde{b}_{jj}}{\widehat{\beta}_{j}}, $$

*where* $\widehat{\beta}_{i}=\tilde{b}_{ii}-\sum_{k=i+1}^{n}|\tilde {b}_{ik}|l_{i}(\tilde{B}^{+})$, *and* $\prod_{j=1}^{i-1}\frac{\tilde{b}_{jj}}{\widehat{\beta}_{j}}=1$ *if* $i=1$.

### Proof

Since *X* is a positive diagonal matrix and $\tilde{M}:=MX$, it is easy to get that $M_{D}= I-D+DM=(X-DX+D\tilde{M})X^{-1}$. Let $\tilde{M}_{D}=X-DX+D\tilde{M}$. Then

$$\tilde{M}_{D}=X-DX+D\tilde{M}=X-DX+D\bigl(\tilde{B}^{+}+ \tilde{C}\bigr)=\tilde {B}_{D}^{+}+\tilde{C}_{D}, $$

where $\tilde{B}_{D}^{+}=X-DX+D\tilde{B}^{+}=[\hat{b}_{ij}]$ with

$$\hat{b}_{ij}=\left \{ \textstyle\begin{array}{l@{\quad}l} x_{i}-d_{i}x_{i}+d_{i}\tilde{b}_{ij}, &i=j,\\ d_{i}\tilde{b}_{ij}, &i\neq j, \end{array}\displaystyle \right . $$

and $\tilde{C}_{D}=D\tilde{C}$. By Lemma 2, $\tilde{B}^{+}$ is strictly diagonally dominant by rows with positive diagonal entries. Similarly to the proof of Theorem 2.2 in [10], we can obtain that $\tilde{B}_{D}^{+}$ is an *SDD* matrix with positive diagonal entries and that

8 $$ \begin{aligned}[b] \big\| M_{D}^{-1}\big\| _{\infty}&\leq \big\| X^{-1}\big\| _{\infty}\cdot\big\| \tilde{M}_{D}^{-1}\big\| _{\infty}\\ &\leq \big\| X^{-1}\big\| _{\infty}\cdot\big\| \bigl(I + \bigl( \tilde{B}^{+}_{D}\bigr)^{-1}\tilde{C}_{D} \bigr)^{-1} \big\| _{\infty}\cdot\big\| \bigl(\tilde{B}^{+}_{D} \bigr)^{-1} \big\| _{\infty}\\ &\leq \max\{\gamma,1\}\cdot(n-1) \cdot\big\| \bigl(\tilde{B}^{+}_{D} \bigr)^{-1} \big\| _{\infty}.\end{aligned} $$

Next, we give an upper bound for $\|(\tilde{B}^{+}_{D} )^{-1} \|_{\infty}$. Notice that $\tilde{B}_{D}^{+}$ is an *SDD* *Z*-matrix with positive diagonal entries, and thus $\tilde{B}_{D}^{+}$ is an *SDD* *M*-matrix. By Lemma 4, we have

$$ \big\| \bigl(\tilde{B}^{+}_{D} \bigr)^{-1} \big\| _{\infty}\leq \sum_{i=1}^{n} \Biggl(\frac{1}{(x_{i}-d_{i}x_{i}+d_{i}\tilde{b}_{ii})(1-u_{i}(\tilde{B}^{+}_{D})l_{i}(\tilde {B}^{+}_{D}))}\prod _{j=1}^{i-1}\frac{1}{1-u_{j}(\tilde{B}^{+}_{D})l_{j}(\tilde {B}^{+}_{D})} \Biggr), $$

where

$$ u_{i}\bigl(\tilde{B}^{+}_{D}\bigr)=\frac{\sum_{j=i+1}^{n}|\tilde {b}_{ij}|d_{i}}{x_{i}-d_{i}x_{i}+\tilde{b}_{ii}d_{i}}, \quad\text{and}\quad l_{k}\bigl(\tilde{B}^{+}_{D}\bigr)=\max _{k\leq i\leq n} \biggl\{ \frac{\sum_{\small\substack{j=k,\neq i}}^{n}|\tilde{b}_{ij}|d_{i}}{x_{i}-d_{i}x_{i}+\tilde{b}_{ii}d_{i}} \biggr\} . $$

By Lemma 5, we deduce for each $k\in N$ that

$$ l_{k}\bigl(\tilde{B}^{+}_{D}\bigr)=\max_{k\leq i\leq n} \biggl\{ \frac{\frac{\sum_{\small\substack{j=k,\neq i}}^{n}|\tilde{b}_{ij}|}{x_{i}}d_{i}}{1-d_{i}+\frac{\tilde {b}_{ii}}{x_{i}}d_{i}} \biggr\} \leq \max_{k\leq i\leq n} \Biggl\{ \frac{1}{\tilde{b}_{ii}}\sum_{\small\substack{j=k,\neq i}}^{n}| \tilde{b}_{ij}| \Biggr\} =l_{k}\bigl(\tilde{B}^{+}\bigr)< 1, $$

and for each $i\in N$ that

9 $$ \begin{aligned}[b] \frac{1}{(x_{i}-d_{i}x_{i}+d_{i}\tilde{b}_{ii})(1-u_{i}(\tilde{B}^{+}_{D})l_{i}(\tilde {B}^{+}_{D}))}&= \frac{1}{x_{i}-d_{i}x_{i}+d_{i}\tilde{b}_{ii}-\sum_{j=i+1}^{n}|\tilde {b}_{ij}|d_{i}l_{i}(\tilde{B}^{+}_{D})} \\ &= \frac{\frac{1}{x_{i}}}{1-d_{i}+\frac{d_{i}}{x_{i}} (\tilde{b}_{ii}-\sum_{j=i+1}^{n}|\tilde{b}_{ij}|l_{i}(\tilde{B}^{+}_{D}) )} \\ &\leq \frac{1}{\min \{\tilde{b}_{ii}-\sum_{j=i+1}^{n}|\tilde{b}_{ij}| l_{i}(\tilde{B}^{+}),x_{i} \}} \\ &=\frac{1}{\min \{ \widehat{\beta}_{i},x_{i} \}}.\end{aligned} $$

Furthermore, according to Lemma 6, it follows that for each $j\in N$,

10 $$ \begin{aligned}[b] \frac{1}{1-u_{j}(B^{+}_{D})l_{j}(B^{+}_{D})}&=\frac{1-d_{j}+\frac{\tilde {b}_{jj}}{x_{j}}d_{j}}{1-d_{j}+\frac{\tilde{b}_{jj}}{x_{j}}d_{j}-\frac{\sum_{k=j+1}^{n}|\tilde{b}_{jk}|}{x_{j}} d_{j} l_{j}(B^{+}_{D})} \\ &\leq\frac{\tilde{b}_{jj}}{\tilde{b}_{jj}-\sum_{k=j+1}^{n}|\tilde{b}_{jk}|l_{j}(\tilde{B}^{+})}=\frac{\tilde {b}_{jj}}{\widehat{\beta}_{j}}.\end{aligned} $$

By (9) and (10), we derive

11 $$ \big\| \bigl(\tilde{B}^{+}_{D} \bigr)^{-1} \big\| _{\infty}\leq \sum_{i=1}^{n} \frac{1}{\min\{\widehat{\beta}_{i},x_{i}\}}\prod_{j=1}^{i-1} \frac{\tilde{b}_{jj}}{\widehat{\beta}_{j}}. $$

Now the conclusion follows from (8) and (11). □

Remark here that when the matrix *M* is a *B*-matrix, then $X=I$ and

$$ \tilde{B}^{+} =[\tilde{b}_{ij}]= \left [ \textstyle\begin{array}{c@{\quad}c@{\quad}c} m_{11}-r_{1}^{+} &\cdots &m_{1n}-r_{1}^{+} \\ \vdots &\ddots &\vdots \\ m_{n1}-r_{n}^{+} &\cdots &m_{nn}-r_{n}^{+} \end{array}\displaystyle \right ], $$

which yields

$$ \max_{d\in[0,1]^{n}}\big\| (I-D+DM)^{-1} \big\| _{\infty} \leq \sum_{i=1}^{n} \frac{n-1}{\min\{\widehat{\beta}_{i},1\}}\prod_{j=1}^{i-1} \frac{\tilde{b}_{jj}}{\widehat{\beta}_{j}}. $$

This upper bound is consistent with that provided by Li *et al.* in [13]. Furthermore, for a $B^{S}$-matrix that is not a *B*-matrix, the following corollary can be obtained easily by Lemma 3 and Theorem 2.

### Corollary 1

*Let* $M=[m_{ij}]\in\mathbb{R}^{n\times n}$ *be a* $B^{S}$-*matrix that is not a* *B*-*matrix*, *and let* $k,i\in N$ *with* $k\neq i$ *such that* $m_{ik}\geq\frac{1}{n}\sum_{j=1}^{n}m_{ij}$. *If* $k\in \overline{S}$, *then*

12 $$ \max_{d\in[0,1]^{n}}\big\| M_{D}^{-1} \big\| _{\infty} \leq \sum_{i=1}^{n} \frac{(n-1)\gamma}{\min\{\widehat{\beta}_{i},1\}}\prod_{j=1}^{i-1} \frac{\tilde{b}_{jj}}{\widehat{\beta}_{j}}; $$

*if* $k\in S$, *then*

13 $$ \max_{d\in[0,1]^{n}}\big\| M_{D}^{-1} \big\| _{\infty} \leq \sum_{i=1}^{n} \frac{n-1}{\min\{\widehat{\beta}_{i},\gamma\}}\prod_{j=1}^{i-1} \frac{\tilde{b}_{jj}}{\widehat{\beta}_{j}}, $$

*where* *γ* *satisfies* (5).

### Example 1

Consider the family of $B^{S}$-matrices for $S=\{1,2\}$:

$$M_{m} = \left [ \textstyle\begin{array}{c@{\quad}c@{\quad}c@{\quad}c} 2 &1 &1 &1.5 \\ -\frac{2m}{m+1} &2 &\frac{1}{m+1} &\frac{1}{m+1} \\ 1 &1 &2 &1 \\ 1 & 1 &1 & 2 \end{array}\displaystyle \right ], $$

where $m\geq1$. Appropriate scaling matrices could be $X=\operatorname{diag}\{\gamma, \gamma, 1, 1\}$, with $\gamma\in(\frac{3.5}{3}, 1.5)$. So $\tilde{M}_{m}:=M_{m}X$ can be written $\tilde{M}=\tilde{B}_{m}^{+}+\tilde{C}_{m}$ as in (3), with

$$ \tilde{B}_{m}^{+} = \left [ \textstyle\begin{array}{c@{\quad}c@{\quad}c@{\quad}c} 2\gamma-1.5 &\gamma-1.5 &-0.5 &0 \\ -\frac{2m}{m+1}\gamma-\frac{1}{m+1} & 2\gamma-\frac{1}{m+1} &0 &0 \\ 0 & 0 &2-\gamma &1-\gamma \\ 0 & 0 &1-\gamma &2-\gamma \end{array}\displaystyle \right ], $$

and

$$ \tilde{C}_{m}=\left [ \textstyle\begin{array}{c@{\quad}c@{\quad}c@{\quad}c} 1.5 &1.5 &1.5 &1.5 \\ \frac{1}{m+1} & \frac{1}{m+1} &\frac{1}{m+1} &\frac{1}{m+1} \\ \gamma & \gamma &\gamma &\gamma \\ \gamma & \gamma &\gamma &\gamma \end{array}\displaystyle \right ]. $$

By computations, we have $\tilde{\beta}_{1}=3\gamma-3.5$, $\tilde{\beta}_{2}=\frac{2(\gamma -1)}{m+1}$, $\tilde{\beta}_{3}=\tilde{\beta}_{4}=3-2\gamma$, $l_{1}({\tilde{B}^{+}})=\max \{\frac{2-\gamma}{2\gamma-1.5},\frac {2m\gamma+1}{2(m+1)\gamma-1},\frac{\gamma-1}{2-\gamma} \}$, $\hat {\beta}_{1}=2\gamma-1.5-(2-\gamma)l_{1}({\tilde{B}^{+}})$, $\hat{\beta}_{2}=2\gamma -\frac{1}{m+1}$, $\hat{\beta}_{3}=\frac{3-2\gamma}{2-\gamma}$, and $\hat{\beta}_{4}=2-\gamma$. Obviously, $M_{m}$ satisfies $m_{ik}\geq\frac{1}{4}\sum_{j=1}^{4}m_{ij}$ for $i=1$ and $k=4$ (∈*S̅*): $1.5>1.375$, which implies that $M_{m}$ is not a *B*-matrix. Then bound (12) in Corollary 1 is given by

$$ 3\gamma \biggl(\frac{1}{\min\{\widehat{\beta}_{1},\gamma\}}+\frac{1}{\min\{ \widehat{\beta}_{2},\gamma\}}\frac{\tilde{b}_{11}}{\widehat{\beta }_{1}}+ \frac{1}{\min\{\widehat{\beta}_{3},\gamma\}}\frac{\tilde {b}_{11}}{\widehat{\beta}_{1}}\frac{\tilde{b}_{22}}{\widehat{\beta }_{2}}+\frac{1}{\min\{\widehat{\beta}_{4},\gamma\}} \frac{\tilde {b}_{11}}{\widehat{\beta}_{1}}\frac{\tilde{b}_{22}}{\widehat{\beta }_{2}}\frac{\tilde{b}_{33}}{\widehat{\beta}_{3}} \biggr), $$

which converges to a constant

$$ 3\gamma \biggl(\frac{1}{3\gamma-3.5}+\frac{2\gamma -1.5}{(3\gamma-3.5)\gamma} +\frac{2(2-\gamma)(2\gamma-1.5)}{(3-2\gamma)(3\gamma-3.5)} \biggr) $$

with $\gamma\in(\frac{3.5}{3}, 1.5)$ when $m\rightarrow+\infty$. In contrast, bound (4) in Theorem 1, with the hypotheses that $m\geq2$, is

$$ \frac{(4-1)\max\{\gamma,1\}}{\min\{\tilde{\beta},\gamma,1\}}=\frac {3\gamma}{2\gamma-1}(m+1) $$

and it can be arbitrarily large when $m\rightarrow+\infty$.

In particular, if we choose $\gamma=1.3$, then bound (4) and bound (7) for $m=2, 20,30,\ldots, +\infty$ can be given as shown in Table 1.

**Table 1:** Bound (4) and bound (7) for $m=2, 20,30,\ldots,+\infty$

*m*	2	20	30	60	100	…	+∞
Bound (4)	**7.3125**	**51.1875**	75.5625	148.6875	246.1875	…	+∞
Bound (7)	48.1089	54.4704	**54.8144**	**55.1699**	**55.3155**	…	**55.5375**

### Remark 1

From Example 1, it is easy to see that each bound (4) or (7) can work better than the other one. This means it is difficult to say in advance which one will work better. However, for a $B^{S}$-matrix *M* with $\tilde{M}= \tilde{B}^{+}+ \tilde{C}$, where the diagonal dominance of $\tilde{B}^{+}$ is weak (e.g., for a matrix $M_{m}$ with a large number of *m* here), we can say that bound (7) is more effective to estimate $\max_{d\in[0,1]^{n}}\|M_{D}^{-1}\|_{\infty}$ than bound (4). Therefore, in general case, for the $\operatorname{LCP}(M, q)$ involved with a $B^{S}$-matrix, one can take the smallest of them:

$$ \max_{d\in[0,1]^{n}}\big\| M_{D}^{-1}\big\| _{\infty} \leq \min \Biggl\{ \frac{(n-1)\max\{\gamma,1\}}{\min\{\tilde{\beta},\gamma,1\}}, \sum_{i=1}^{n} \frac{(n-1)\max\{\gamma,1\}}{\min\{\hat{\beta}_{i},x_{i}\} }\prod_{j=1}^{i-1} \frac{\tilde{b}_{jj}}{\hat{\beta}_{j}} \Biggr\} . $$

To measure the sensitivity of the solution of the *P*-matrix linear complementarity problem, Chen and Xiang in [5] introduced the following constant for a *P*-matrix *M*:

$$ \beta_{P}(M)=\max_{d\in[0,1]^{n}}\big\| (I-D+DM)^{-1}D \big\| _{P}, $$

where $\|\cdot\|_{p}$ is the matrix norm induced by the vector norm for $p \geq1$.

Similarly to the proof of Theorem 2.4 in [1], we can also give new perturbation bounds for $B^{S}$-matrices linear complementarity problems based on Theorem 2.

### Theorem 3

*Let* $M=[m_{ij}]\in\mathbb{R}^{n\times n}$ *be a* $B^{S}$-*matrix and* $\tilde{B}^{+}=[\tilde{b}_{ij}]$ *be the matrix given in Lemma* 2. *Then*

$$ \beta_{\infty}(M)\leq \sum_{i=1}^{n} \frac{(n-1)\max\{\gamma,1\}}{\min\{\widehat{\beta }_{i},x_{i}\}}\prod_{j=1}^{i-1} \frac{\tilde{b}_{jj}}{\widehat{\beta}_{j}}, $$

*where* $\widehat{\beta}_{i}=\tilde{b}_{ii}-\sum_{k=i+1}^{n}|\tilde {b}_{ik}|l_{i}(\tilde{B}^{+})$, *and* $\prod_{j=1}^{i-1}\frac{\tilde{b}_{jj}}{\widehat{\beta}_{j}}=1$ *if* $i=1$.

Similarly, by Corollary 1 and Theorem 3, we can derive the following corollary.

### Corollary 2

*Let* $M=[m_{ij}]\in\mathbb{R}^{n\times n}$ *be a* $B^{S}$-*matrix that is not a* *B*-*matrix*, *and let* $k,i\in N$ *with* $k\neq i$ *such that* $m_{ik}\geq\frac{1}{n}\sum_{j=1}^{n}m_{ij}$. *If* $k\in \overline{S}$, *then*

$$ \beta_{\infty}(M)\leq \sum_{i=1}^{n} \frac{(n-1)\gamma}{\min\{\widehat{\beta}_{i},1\}}\prod_{j=1}^{i-1} \frac{\tilde{b}_{jj}}{\widehat{\beta}_{j}}; $$

*if* $k\in S$, *then*

$$ \beta_{\infty}(M)\leq \sum_{i=1}^{n} \frac{n-1}{\min\{\widehat{\beta}_{i},\gamma\}}\prod_{j=1}^{i-1} \frac{\tilde{b}_{jj}}{\widehat{\beta}_{j}}, $$

*where* *γ* *satisfies* (5).

## Conclusions

In this paper, we give an alternative bound for $\max_{d\in [0,1]^{n}}\|(I-D+DM)^{-1}\|_{\infty}$ when *M* is a $B^{S}$-matrix, which improves that provided by García-Esnaola and Peña [1] in some cases. We also present new perturbation bounds of $B^{S}$-matrices linear complementarity problems.
